# American Academy of Dental Science

**Published:** 1892-08

**Authors:** 

**Affiliations:** American Academy of Dental Science


### Reports of Society Meetings.

### AMERICAN ACADEMY OF DENTAL SCIENCE.

THE regular monthly meeting of the American Academy of Dental Science was held at the Boston Medical Library Association rooms on Wednesday, March 2, 1892, at 7.30 P.M., President Brackett in the chair.

The paper of the evening was by Dr. Louis Jack. Subject, "The Fixation of Dental Matrices and the Packing of the Cervical Portion of the Cavities."

(For Dr. Jack's paper, see page 565.)

### DISCUSSION.

*Dr. Meriam.*—There are many of our younger members who ought to know that to Dr. Jack we owe the matrix. After Dr. Jack made it known, not much was heard of it till about five or eight years ago. It then gained prominence, perhaps on account of Dr. Herbst's methods, and our shops and our offices were flooded with many forms of this device. Dr. Jack presented the matrix to the profession without patenting it (it was the trading element that did that later), and it is to him we owe it as a professional instrument. You can see by the paper to-night that the fixation of the matrix requires care and precision. In the hands of Dr. Jack the matrix reduces the filling of teeth almost to an exact science, but the probability is that it saves little time except in the finishing.

*Dr. Fillebrown.*—I learned the art of filling teeth without the use of the matrix, and I have been inclined to operate without it ever since. I have, however, used the matrix enough to know that it is a very valuable help in many cavities. It needs to be used with a good deal of discretion and care. The matrix must be considered as an important wall of the cavity, and the filling must be as carefully adapted to it as to the other walls. Unless you do, the filling is soft at the cervix and imperfect at the margins. We have found it a difficult thing to get students to use the matrix with skill. While the matrix that was invented by Dr. Jack and presented here to-night in connection with this paper is a valuable device, there are various improved forms in use to-day, and among them might be mentioned the Ladmore-Brunton. This is a steel band which encircles the tooth. The steel is thin enough to go between teeth that are very close together. The band is tightened around the tooth by means of screws.

*Dr. Taft.*—I have found the matrix especially valuable in compound fillings. The one I use is made of silvered copper. It is secured in place by a ligature passed through two holes punched through either end.

*President Brackett.*—Dr. Jack is not confined to the use of a matrix in the filling of corono-approximal cavities. This is one of his resources.

*Dr. Ainsworth.*—I never think of filling an approximal cavity in a bicuspid or molar, where the coronal portion is involved, without a matrix. After investigating all forms of matrices, I now use thin cold-rolled steel, which can be bent, and yet retain, to a certain degree, its elasticity. The Perry separator is an excellent device for holding a matrix is position. A piece of orange-wood can be adjusted at the cervical wall. To my mind, the matrix saves time not only in finishing, but all through the operation. It also enables one to build up a bicuspid badly gone on both mesial and distal sides, and for this purpose a band of German silver completely encircling the tooth is very convenient. It can be held in position by packing cement on both sides.

I have never taken up combination fillings of gold and amalgam, because with the matrix I can use soft foil quite as rapidly as amalgam, and with more satisfactory results. And it is very rare for fillings so made to give out.

*Dr. Taft.*—I cannot quite agree with Dr. Ainsworth that soft foil can be used as rapidly as amalgam in the cavities under consideration. As to matrices, silvered copper has a decided advantage for me, in that it lights up the cavity.

*Dr. H. A. Baker.*—I have probably used the matrix as much as any man in New England. I was the first one in New England to adopt it after Dr. Jack made it known, and I was also the first one to introduce the copper matrix, and then the silvered copper. I am using at present a thin steel matrix, and the Perry separator for fixing it.

*Dr. Wilson.*—I would like to ask Dr. Baker if he uses an absolutely soft gold with the matrix—a gold that cannot be made cohesive?

*Dr. Baker.*—I am now using an unannealed cohesive gold number three foil.

*Dr. Meriam.*—In England they usually speak of such gold as stale gold. It seemed a good word to adopt in distinguishing it from absolutely non-cohesive gold.

*Dr. Adams.*—There is one use of the matrix which has given me a great deal of satisfaction, and that is in very large buccal cavities in the lower molars which are to be filled with amalgam. Such cavities extending below the gum are very difficult to treat. I use a matrix of thin steel, concaving it with a round-faced hammer on a block of lead, then bending it around the tooth and tying with ligatures. This matrix is applied before the cavity is excavated, and after it is applied the rubber dam is adjusted. The matrix fits closely to the tooth and becomes a guide in excavating, and, if properly fitted, excludes all moisture.

*Dr. H. A. Baker.*—Have you filled such cavities where they extend beneath the gum?

*Dr. Adams.*—Yes. I do not use the matrix unless the cavity does go beneath the gum. I can carry the matrix down to the bifurcation of the roots, and it is a great assistance in excavating. With amalgam, of course, a very thin steel can be used, because there is not much pressure. The holes in the matrix are made quite low down, and the ligature is wound several times around the tooth.

*Dr. Meriam.*—The ring steel matrix can be used with great advantage in the case of badly-decayed molars requiring the restoration of more than one surface. The steel can be fitted as a ring, and soldered and left in the mouth until an amalgam filling has hardened. There is no more difficulty in soldering cold rolled steel than in soldering German silver.

*Dr. Adams.*—I wish to present a cast showing the cuspid situated between the two bicuspids.

*Dr. Ainsworth.*—I have been trying, to a limited extent, the method about which Dr. Clapp spoke at one of our meetings, of depending upon cement to retain gold in cavities where it is difficult to gain an undercut. I think highly of the method, and have been able to make several fillings which have seemed entirely satisfactory.

*Dr. Robinson.*—Just after Dr. Clapp mentioned the method alluded to I tried it in mesial cavities in bicuspids and molars, and also in frail centrals, and the results have been very satisfactory.

*Dr. Williams.*—Several years ago Dr. Buckland advocated a combination of cement and amalgam in cavities with frail walls. I used this principle, and from it the idea came to me of embedding crystal gold in cement while soft, and thus gaining a foundation for a gold filling.

*Dr. Fillebrown.*—More than twenty years ago cavities which were so large and with such small undercuts that it was useless to think of putting gold-foil in them I filled with oxychloride and allowed it to harden, and then cut grooves in it and filled over it with gold; such fillings did good service for many years.

*Dr. Meriam.*—It is about fifteen years since Dr. Kidder read a paper advocating the use of Canada balsam in connection with Watts's crystal gold for starting a filling.

*President Brackett.*—I had a case of a cavity in a lower incisor, involving the cutting-edge and distal surface. The extremely frail walls of the cavity rendered it impossible to make suitable retaining cuts, and I tried this method of putting Steurer's gold into soft cement and building on that as a foundation. The filling was completed, but inadvertently left prominent, so that for a week it was subjected to the extra strain of undue occlusion; yet it was not displaced. Since dressing it to proper contour several weeks have passed and it has remained absolutely undisturbed.

*Dr. Eddy.*—There is a preparation of gold made by the S. S. White Dental Manufacturing Company similar to Steurer's gold, but not as friable. It comes in small mats, and there is not so much waste as with Steurer's.

*President Brackett.*—If there is no objection, we come to Dr. Stoddard's presentation of a new dental lamp-fixture.

*Dr. Stoddard.*—I have been using this electric lamp for the past two or three months with considerable satisfaction. It is intended to be attached to an Allen table, and the wires can be easily connected with an ordinary electric-light socket. The fixture is pivoted to this brass plate, which is screwed on to the under part of the table. It can be moved forward or backward, and is easily adjusted. Vertical motion is supplied by the bracket. When it is not in use, it can be turned around out of the way. I use a sixteen candle-power Edison lamp, which is rather stronger than the S. S. White lamp, which, I believe, is but five candle-power. The lamp is cylindrical in form, and the shade is similar to the pulpit shade, and is perfectly adjustable. There is comparatively little heat from the light.

*President Brackett.*—I have seen this lamp in use, and it certainly seems a very efficient illuminant and free from the many objections to gas.

William H. Potter, D.M.D.,
*Editor American Academy of Dental Science.*

